# The Experience of Breastfeeding Women During the Pandemic in Romania

**DOI:** 10.3390/children12101279

**Published:** 2025-09-23

**Authors:** Ruxandra-Gabriela Cigăran, Gheorghe Peltecu, Radu Botezatu, Nicolae Gică

**Affiliations:** 1 Carol Davila University of Medicine and Pharmacy, 020021 Bucharest, Romania; ruxandra-gabriela.cigaran@drd.umfcd.ro (R.-G.C.); gheorghe.peltecu@gmail.com (G.P.); radu.botezatu@umfcd.ro (R.B.); 2 Department of Obstetrics and Gynecology, Filantropia Clinical Hospital, 011171 Bucharest, Romania

**Keywords:** breastfeeding, COVID-19, pandemic, mental health, healthcare

## Abstract

**Highlights:**

**What are the main findings?**

• The pandemic posed an additional psychological burden for women who breastfed during this period.
• Despite elevated stress, mothers demonstrated a persistent commitment to breastfeeding; access to breastfeeding support during the pandemic remained generally limited.

**What are the implications of the main findings?**

• Emphasizing the essential role of psychological support for breastfeeding mothers during the pandemic, facilitating both the initiation and the continuation of breastfeeding in the context of public health crises.
• Highlighting the need to identify effective interventions to support mothers during public health crises.

**Abstract:**

**Background:** Evidence suggests that the uncertainty surrounding the COVID-19 pandemic and the associated changes have negatively influenced breastfeeding practices. Considering that breastfeeding women are already known to be more vulnerable in terms of emotional status, the aim of our study was to evaluate the experiences and concerns of breastfeeding women in Romania during the pandemic. Also, we tried to identify the most effective measures for mitigating the negative impacts. **Methods:** A 46-item questionnaire was developed for data collection and it was shared on Facebook and Instagram, with networks of women who breastfed during the pandemic and with obstetric communities. Our cross-sectional survey recruited 261 Romanian breastfeeding women. Among general questions about basic demographic information and details about changes experienced during the COVID-19 pandemic, the survey included questions about their breastfeeding experiences during the pandemic, whether they had contracted SARS-CoV-2 while breastfeeding, their concerns, and their overall perceptions of the period. **Results:** In our study, we observed that women who breastfed during the pandemic—especially those who had contracted SARS-CoV-2—experienced significant fear that their newborns or children might become infected. These mothers also expressed deep concern for their own health and the wellbeing of their loved ones. The experience of contracting SARS-CoV-2 was a major source of psychological stress. Despite these challenges, the participants, especially women who contracted SARS-CoV2, reported a notably longer overall duration of breastfeeding and higher rates of exclusive breastfeeding. However, access to breastfeeding support during this period was generally limited. **Conclusions:** These findings highlight the negative impact of the pandemic on breastfeeding mothers and the adaptability of mothers under crisis conditions, emphasizing the need for improved support systems and targeted interventions to assist mothers during public health crises.

## 1. Introduction

In March 2020, the World Health Organization (WHO) officially declared COVID-19 a global pandemic [1]. As is now well known, COVID-19 is caused by the SARS-CoV-2 virus (severe acute respiratory syndrome coronavirus 2), first identified in China at the end of 2019 [1,2]. Like many other countries around the world, Romania was also impacted by the pandemic, with the first confirmed case reported on 26 February 2020 [3]. The SARS-CoV-2 pandemic significantly impacted the global economy, national infrastructure, healthcare systems and the general wellbeing of populations worldwide [4,5]. Since the virus spreads primarily through respiratory droplets and close contact, various public health measures were introduced globally to prevent its transmission, including lockdowns and social distancing [4,5,6]. In response to the evolving situation, the Romanian authorities implemented a range of measures aimed at protecting public health and adapting to the challenges brought on by the pandemic [6].

The WHO advocates for exclusive breastfeeding from birth up to at least six months of age and ideally continuing until the child reaches two years of age [7,8]. Breastfeeding plays a vital role in ensuring optimal nutrition, supporting healthy growth and development in infants [7,8]. It supplies essential antibodies that help protect against infectious diseases, reduces the risk of obesity and has been associated with enhanced cognitive development [8]. Mothers also benefit significantly from breastfeeding, including a reduced risk of developing breast and ovarian cancers [8]. Given these wide-ranging advantages, promoting and supporting breastfeeding is of critical importance.

Although studies have demonstrated that expressed breast milk from mothers infected with COVID-19 is safe for their newborns [9,10,11], some researchers advised against direct breastfeeding [12]. They recommended isolating SARS-CoV-2-positive mothers from their infants [12]. As a result, key practices such as mother–infant bonding, rooming-in, and breastfeeding became major concerns among mothers with confirmed COVID-19 infections [13].

Breastfeeding is a multifaceted practice influenced by both personal and environmental factors [13]. The COVID-19 pandemic introduced considerable uncertainty, especially concerning the impact of SARS-CoV-2 infection during the peripartum period [13,14]. This uncertainty adversely affected maternal mental health, with new mothers becoming particularly susceptible to emotional and psychological stress [13,15]. As a result, the disruptions caused by the pandemic present substantial risk factors that may hinder both the initiation and continuation of breastfeeding [16,17].

Hospitals revised their labor and delivery protocols to prioritize the safety of both mothers and newborns, which included limiting the number of visitors and shortening hospital stays [18]. Additionally, numerous government directives were implemented during the pandemic to help contain the spread of the virus [18]. One notable consequence of pandemic-related protocols was the separation of mothers and newborns immediately after birth, particularly in cases where the mother tested positive for or was suspected of having COVID-19 [12]. These measures disrupted early mother–infant bonding and often impeded opportunities for breastfeeding. Such interventions are expected to have a negative impact on maternal mental health and highlight significant challenges in formulating appropriate policies and recommendations within maternal healthcare systems [13,14,15,16].

Although no evidence has shown an increased risk of neonatal infection during vaginal delivery [19], most clinical guidelines during the pandemic favored cesarean sections as the preferred mode of birth [20]. As a result, the rate of C-sections rose significantly [20]. According to a study analyzing data from 191 Romanian hospitals (171 public and 20 private), the cesarean section rate in 2020 stood at 52.9%, a figure that greatly exceeded the WHO’s recommended rate of 10–15%. While this study does not provide a direct pre-pandemic versus pandemic comparison, it highlights a concerning trend of elevated C-section rates during the pandemic period [21]. However, the method of delivery—whether vaginal or cesarean—is an important factor influencing the initiation of breastfeeding [13]. Considering the higher rates of cesarean deliveries among COVID-19-positive mothers, it is essential to provide adequate support for both the initiation and continuation of breastfeeding [13].

Prior to the COVID-19 pandemic, breastfeeding support in Romania was limited. National programs promoted exclusive breastfeeding in hospitals and primary care settings, but access to professional lactation consultants was uneven, and it was most concentrated in urban areas [22]. Ongoing support postpartum was limited, contributing to relatively short durations of exclusive breastfeeding [22]. Community support, such as peer networks or lactation counseling beyond hospital settings, remained underdeveloped.

Research has shown that the COVID-19 pandemic influenced breastfeeding practices through a combination of personal, psychological, environmental and social factors [13,15]. The duration and overall experience of breastfeeding may have been influenced by the circumstances surrounding the COVID-19 pandemic [13,15]. While a few studies have addressed appropriate care for pregnant and postpartum women during the COVID-19 pandemic [18,23], limited attention has been given to the breastfeeding experiences of mothers in Romania during this period. Therefore, this study aims to explore how Romanian mothers experienced breastfeeding throughout the COVID-19 pandemic. In order to better understand the impact of COVID-19 on maternal experiences, this study compared two groups of breastfeeding women: those who were infected with SARS-CoV-2, and those who were not. This distinction was central to our design, as it allowed us to examine whether infection status influenced breastfeeding duration, exclusivity, emotional wellbeing, and sources of support.

A key objective was to gain insight into the changes brought on by the pandemic, which impacted breastfeeding mothers and heightened their concerns. We hope that our findings will contribute to strengthening support for this vulnerable group and guiding improvements in maternity care—both during public health crises and beyond—in order to minimize adverse effects.

## 2. Materials and Methods

### 2.1. Design

A 46-item questionnaire was developed for data collection, targeting Romanian women who breastfed during the COVID-19 pandemic. Participants were self-recruited by completing the questionnaire, which was distributed via Facebook, Instagram, and obstetrics-focused communities. The survey, available online in Romanian, was created using Google Forms by a multidisciplinary team of obstetricians and psychiatrists. We obtained the approval of the Ethics Council of Filantropia Hospital, Bucharest (No.4.1/31 July 2025).

The questionnaire consisted primarily of closed-ended and multiple-choice questions. A pilot study was not conducted; however, the questionnaire was reviewed by a multidisciplinary team to ensure clarity and content validity before dissemination. Participants provided basic demographic information and details about changes experienced during the COVID-19 pandemic. They were also asked about their breastfeeding experiences during the pandemic, whether they had contracted SARS-CoV-2 while breastfeeding, their concerns and their overall perceptions of the period. Emotional and psychological variables were assessed using a set of ad hoc questions specifically developed for this study. The items were designed collaboratively by a multidisciplinary team consisting of obstetricians and psychiatrists, with the goal of capturing maternal experiences related to breastfeeding during the COVID-19 pandemic. This tailored approach allowed us to address issues relevant to the Romanian context and the unique circumstances of the pandemic. These responses aimed to assess the experience and the emotional and psychological impacts of the pandemic on breastfeeding mothers.

### 2.2. Participants

A total of 261 self-identified women who breastfed during pandemic were recruited via Facebook and Instagram, including breastfeeding-specific groups. The questionnaire link was also shared with medical communities for distribution through their networks of women who breastfed during the pandemic. Eligible participants were Romanian women aged ≥18 years who were breastfeeding during the COVID-19 pandemic. The inclusion criteria were as follows: breastfeeding a child during the pandemic period, residing in Romania during the pandemic, and willingness to complete the study questionnaire. The exclusion criteria included women who did not provide complete questionnaire responses.

Participants were divided into two groups based on self-reported COVID-19 infection status confirmed by a positive PCR or antigen test: the experimental group included women who had been infected with SARS-CoV-2 while breastfeeding, and the control group included women who had not been infected during the same period.

### 2.3. Analysis of Data

Data analysis was performed in Python 3.7 using Pandas [24] DataFrames for data selection and descriptive statistics, SciPy [25] for chi-squared analyses, and matplotlib for data visualization [26]. *p*-values of less than 0.05 were considered statistically significant, and throughout the manuscript the degree of significance was codified as follows: * *p* < 0.05, ** *p* < 0.01, *** *p* < 0.001

## 3. Results

The 261 respondents were divided into groups based on whether they contracted SARS-CoV-2 during the breastfeeding period (SARS-CoV-2) or not (control). To explore how maternal SARS-CoV-2 infection influenced breastfeeding experiences during the pandemic, we compared an experimental group of women who had been infected with COVID-19 while breastfeeding with a control group of women who were not infected. This comparison allowed us to differentiate challenges and coping strategies that were specific to infection from those that were more broadly experienced due to pandemic-related disruptions, such as social isolation, limited access to healthcare, and changes in daily routines. By including a control group, we aimed to identify whether infection introduced unique barriers to breastfeeding or whether the observed experiences reflected general stressors affecting all mothers during this period. This approach enhances the interpretability of our findings and provides evidence to guide targeted support for both infected and non-infected breastfeeding women in public health contexts.

The study included 261 participants, with 125 in the SARS-CoV-2 group and 136 in the control group. Among all participants, 54% were between 26 and 35 years old, and the majority had a higher education degree (92.3%) (Table 1). In terms of breastfeeding practices during the pandemic, 81.6% of the total sample reported exclusive breastfeeding. When comparing groups, 86.4% of mothers in the SARS-CoV-2 group exclusively breastfed, compared to 77.2% in the control group. We also observed that, across the entire sample, the majority of mothers breastfed for more than one year (Table 1).

**Figure 1:**
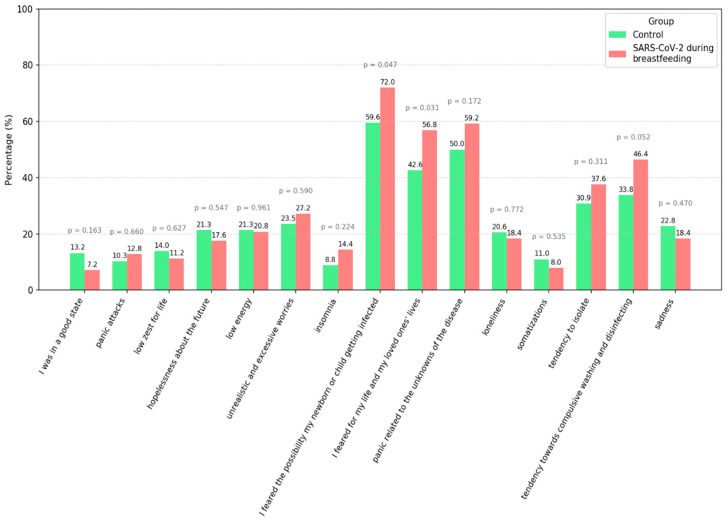
Percentages of respondents experiencing various emotional states and symptoms.

A small number of respondents reported being in a good state and, although not statistically significant, the percentage of respondents in the SARS-CoV-2 group reporting being in a good state was half that of respondents in the control group (7.2% vs. 13.2%, *p* = 0.163, chi-squared test). Significantly higher percentages of patients in the SARS-CoV-2 group compared to the control group experienced fear of the possibility that their newborn/child might contract the disease (72% vs. 59.6%, respectively, *p* = 0.047 *, chi-squared test) and fear for their own or their loved ones’ lives (56.8% vs. 42.6%, *p* = 0.031 *, chi-squared test) (Figure 1, Table 2).

**Figure 2:**
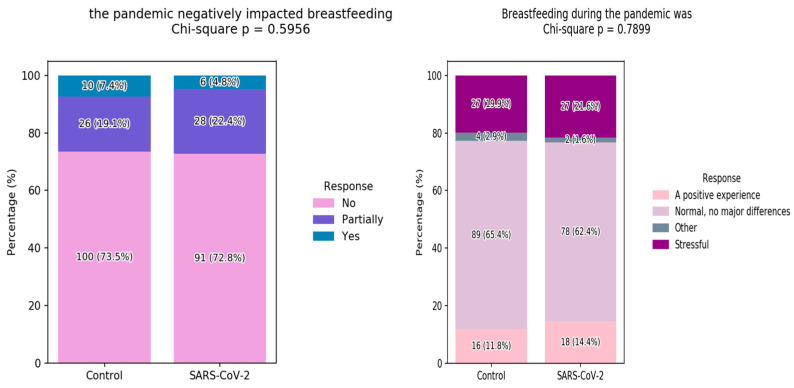
The influence of the pandemic over the breastfeeding process.

Virtually no differences were observed in the perceived influence of the pandemic over the breastfeeding process, with about 73% of respondents in both groups assessing that the SARS-CoV-2 pandemic had no negative impact on their breastfeeding. SARS-CoV-2 infection during breastfeeding increased the percentage of breastfeeding being reported as stressful by a negligible 1.7% (21.6%, from the 19.9% in the control group). However, slightly more women in this group also reported breastfeeding being a positive experience (14.4% vs. 11.8%), and no statistically significant differences were observed by the chi-squared tests (Figure 2).

**Figure 3:**
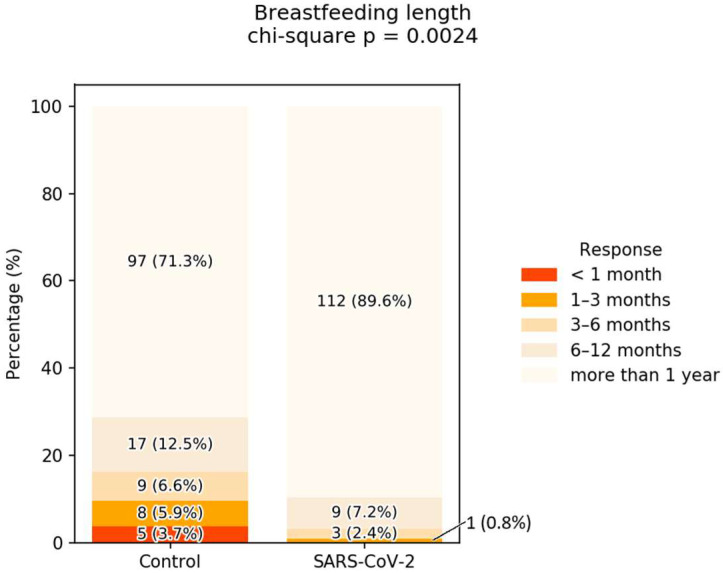
The breastfeeding duration.

Surprisingly, women who contracted SARS-CoV-2 during breastfeeding reported significantly longer total breastfeeding, with 89.6% breastfeeding for more than 1 year, compared with only 71.3% of women in the control group. This high percentage was associated with a reduction in the number of women breastfeeding for less than 6 months, from 16.2% in the control group to only 3.2% in the SARS-CoV-2 group (the overall *p*-value observed in the chi-squared test for breastfeeding duration was 0.0024 **) (Figure 3).

**Figure 4:**
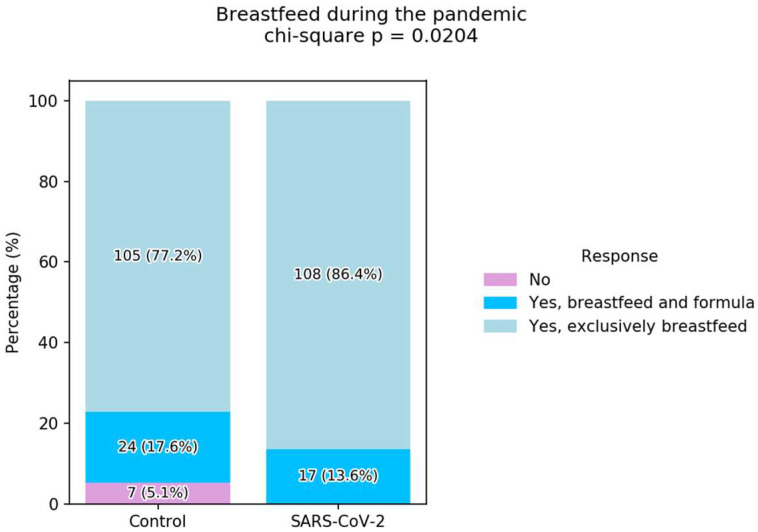
Breastfeeding during the pandemic.

Significantly more women who were infected with SARS-CoV-2 reported feeding their child exclusively by breastfeeding compared to women who were not infected (86.4% vs. 77.2%, *p* = 0.0204, chi-squared test) (Figure 4).

**Figure 5:**
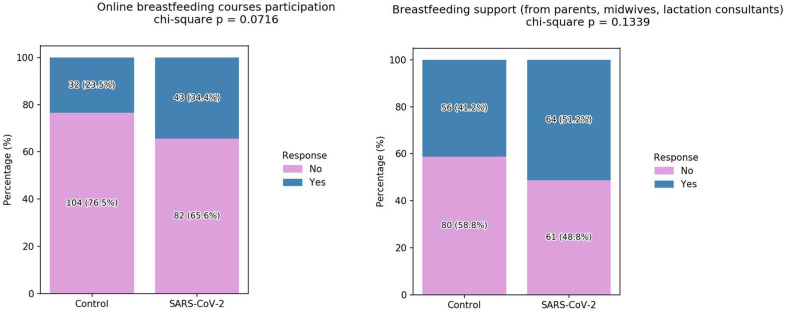
Sources of breastfeeding support during the COVID-19 pandemic. Left panel: online resources (participation in online breastfeeding courses). Right panel: interpersonal support (family members, midwives, and lactation consultants).

Breastfeeding support from parents, midwives, lactation consultants, or online courses was infrequently reported within our cohort; however, it was slightly more prevalent among women who had contracted SARS-CoV-2 (Figure 5). When examining sources of support, two distinct patterns emerged. Participation in online breastfeeding courses was reported by 34.4% of infected mothers, compared with 23.5% in the control group (*p* = 0.0716). In contrast, reliance on family and professional support (parents, midwives, lactation consultants) was reported by 51.2% of infected mothers and 41.2% of controls (*p* = 0.1339). Although these differences did not reach statistical significance, the findings suggest that both online and interpersonal resources played a role in sustaining breastfeeding during the pandemic (Figure 5).

## 4. Discussion

This study explored first-time mothers’ experiences with breastfeeding during the COVID-19 pandemic in Romania. Women giving birth and breastfeeding during the COVID-19 outbreak had to cope with new challenges in addition to the usual stress of being a new mother. Overall, mothers reported both challenges and benefits associated with breastfeeding during the pandemic. Our analysis was centered on comparing infected and non-infected mothers, as infection status represented a key variable potentially shaping both breastfeeding practices and maternal psychological experiences during the pandemic.

It is also described in the literature that the pandemic has brought both positive and negative impacts on breastfeeding mothers, often leading to complex emotional responses [27,28,29,30,31]. In particular, many mothers have experienced heightened feelings of guilt, anxiety, and depression [27]. These emotional challenges may pose potential risks to successful breastfeeding [27,28,29,30,31].

Several factors were identified as negatively influencing breastfeeding during the COVID-19 pandemic, including mother–infant separation, lack of skin-to-skin contact, inadequate support, reliance on online breastfeeding resources, and fear related to the pandemic and of contracting infection [13,27,28,29,30,31].

Breastfeeding women during the pandemic reported experiencing several negative emotions, including fear of contracting the virus, concerns about being unable to breastfeed or potentially infecting their baby, and the emotional toll of loneliness and limited social support [13,31]. Many also felt sadness and inadequacy, struggled with understanding social isolation measures, faced challenges in adhering to hygiene protocols, and expressed a strong need for information, guidance, and increased support from others [31].

In our study, participants—particularly those who had experienced a SARS-CoV-2 infection—reported a strong fear that their newborn or child might contract the virus, along with concerns for their own lives and the lives of their loved ones. Other emotions, such as panic stemming from the uncertainties of the disease, as well as a tendency toward compulsive washing, disinfecting, and self-isolation, were reported among the women—although these feelings were more intense among those who had contracted COVID-19. Although most of the respondents did not perceive the pandemic itself as having a negative impact, contracting SARS-CoV-2 was a significant source of stress for mothers. Our findings align with patterns reported in other international contexts. For example, a study from Saudi Arabia found that many mothers expressed concerns about the risk of contracting COVID-19 and transmitting it to their newborns. At the same time, mothers described the pandemic as a period that reinforced their commitment to breastfeeding and strengthened the mother–infant bond [13].

An interesting finding of our study was that women who were infected with COVID-19 during breastfeeding reported a significantly longer duration of breastfeeding and higher rates of exclusive breastfeeding compared to non-infected mothers. We observed that, in our group, a significantly longer overall breastfeeding duration was reported. Among all participants, 80.1% breastfed for more than one year, with an even higher percentage observed among women who had contracted SARS-CoV-2. Although the underlying reasons remain unclear, several contextual factors in Romania may contribute to this pattern. Infected mothers may have considered breast milk to be a crucial source of immune protection for their infants, reinforcing their commitment to prolonged breastfeeding. Additionally, infection-related isolation and limited access to alternative feeding options, such as formula or in-person lactation support, may have unintentionally encouraged continued breastfeeding. An Israeli cross-sectional study found that many mothers extended their breastfeeding period during lockdown largely because they stayed home longer than expected, particularly when returning to work was delayed [32]. Chien et al. demonstrated that breastfeeding intentions and behaviors remained high during the COVID-19 pandemic, and that online support from health professionals proved to be effective during this period [28]. This can be seen as one of the few positive outcomes of the pandemic. Considering our findings, the pandemic did not negatively impact the overall duration of breastfeeding. These observations highlight a potentially important behavioral response to maternal illness that warrants further investigation. Future studies, particularly qualitative research exploring mothers’ motivations and decision-making processes, could clarify these mechanisms and inform public health strategies supporting breastfeeding during health crises.

Our results revealed an apparent paradox: mothers who were infected with SARS-CoV-2 reported elevated psychological stress but also longer breastfeeding durations. One possible explanation is that breastfeeding during the pandemic carried a dual role—acting both as a stressor and as a coping strategy. On the one hand, mothers faced heightened anxiety, limited support, and uncertainty regarding infection risks, all of which could elevate psychological distress. On the other hand, breastfeeding may have been perceived as one of the few available ways to actively protect infants during a time of crisis, reinforcing maternal motivation to continue despite the difficulties. Similar dynamics have been described in international studies, where mothers reported that stress and anxiety coexisted with a stronger commitment to sustain breastfeeding as a protective measure for the child [33,34]. This suggests that in contexts of heightened uncertainty, maternal perseverance with breastfeeding may reflect not the absence of stress but, rather, a deliberate decision to endure stress for the perceived benefit of the infant. Breastfeeding may serve as both a buffer against psychological distress and a proactive strategy to nurture and protect the infant under crisis.

Another widely discussed issue during the pandemic was the lack of adequate support for breastfeeding women. Many professional lactation care providers were reassigned to other healthcare roles, and lockdowns significantly limited or eliminated face-to-face support from peers and family. As a result, many new mothers were left without sufficient guidance or assistance. Although online support was available, it often fell short of meeting mothers’ needs [29,30,31]. Badr et al. emphasized that family support was a critical factor in sustaining breastfeeding during the pandemic [13], while a study from Spain reported that pandemic-related disruptions resulted in unequal access to professional support and greater reliance on online peer networks [35]. In our study, breastfeeding support was generally limited. Only 46% of all respondents reported receiving any breastfeeding support—from parents, midwives, or lactation consultants—while just 28.7% preferred online breastfeeding courses. However, some studies also highlighted unexpected benefits of the pandemic period, such as increased time for bonding with the baby and the opportunity to breastfeed without the distractions of visitors [29,30,32]. The restrictions on access to social support systems and the widespread isolation measures implemented during the pandemic had a broad impact on the general population [5,6]. However, pregnant and postpartum women are known to be particularly vulnerable in terms of psychological wellbeing, and they may have been disproportionately affected during this period [18,23]. Healthcare providers should engage in more frequent counseling with new mothers and their families during the postpartum period, emphasizing the promotion of breastfeeding, preventive measures against COVID-19 infection, and the initiation and maintenance of mother–infant bonding after birth [18,20,23,27,29,31].

During the pandemic, it was essential to implement and strictly adhere to general infection control measures to prevent neonatal infection. However, these precautions may have contributed to increased stress for new mothers. Several studies recommend that healthcare professionals support breastfeeding women and their families during the pandemic through targeted educational and therapeutic interventions [27,29,31].

As we observed, our findings highlight the dual challenges faced by Romanian mothers during the COVID-19 pandemic: elevated stress levels alongside sustained breastfeeding commitment, particularly among those who were infected. These results underscore the importance of strengthening support systems that can simultaneously address lactation needs and maternal psychological wellbeing. International evidence points to several effective strategies, including online services that reduce early cessation of breastfeeding [36,37], integration of mental health screening into routine postpartum care as recommended by the WHO [38], and the inclusion of breastfeeding in emergency preparedness plans [39].

Building on these insights, we outline context-specific recommendations for Romania that aim to strengthen resilience in maternal and child health services during both ordinary times and future crises.

## 5. Conclusions

In conclusion, the pandemic posed an additional psychological burden for women who breastfed during this period. Their primary concerns centered around potential threats to their own health and that of their infants, largely due to the uncertainties surrounding SARS-CoV-2 and the evolving nature of the pandemic. SARS-CoV-2 infection was a significant source of stress for these mothers. On a positive note, a notably longer overall duration of breastfeeding was observed, although access to breastfeeding support during this period remained generally limited. Our findings reveal that mothers infected with SARS-CoV-2 not only sustained breastfeeding but reported significantly longer breastfeeding durations and higher rates of exclusive breastfeeding compared with non-infected women. This result is especially striking given the elevated psychological stress observed among infected mothers, suggesting that breastfeeding may have functioned both as a protective strategy for infants and as a coping mechanism for mothers. The comparison between infected and non-infected mothers shows that COVID-19 infection status significantly shaped both breastfeeding practices and maternal psychological wellbeing, adding context-specific evidence from Romania.

By documenting these dynamics in Romania, our study fills a critical gap in understanding how pandemic-related disruptions shaped maternal practices in a setting with limited pre-pandemic breastfeeding support infrastructure. These findings emphasize the resilience and adaptability of mothers under crisis conditions, while also pointing to the urgent need for stronger structural support, including online lactation counseling, integrated psychological care, and preparedness planning for future public health emergencies.

Ultimately, this research highlights not only the challenges but also the perseverance of breastfeeding mothers during the pandemic, offering lessons for strengthening maternal–infant health systems in Romania and comparable contexts worldwide.

Further research is warranted to explore the effects of the pandemic on breastfeeding practices and infant health outcomes, and to identify effective interventions to support mothers during such public health crises.

## 6. Limitations

Several limitations of this study should be noted. First, the cross-sectional design precludes causal inference between maternal experiences, SARS-CoV-2 infection status, and breastfeeding outcomes. Second, recruitment via social media likely resulted in a non-representative sample, with over-representation of younger, urban, and highly educated women, which may limit the generalizability of our findings. Moreover, the participants were auto-recruited, and the researchers were not able to ask further questions to clarify the responses. Third, eligibility criteria for specific subgroups, such as mothers of multiples, preterm infants, or women no longer breastfeeding, were not explicitly defined, limiting the interpretability of subgroup-specific results. Fourth, emotional and psychological variables were assessed using ad hoc questions rather than validated instruments, which may reduce the reliability and comparability of these findings. Finally, our analyses were limited to bivariate comparisons (e.g., chi-squared tests). We did not conduct multivariate models controlling for socio-demographic and contextual factors, which means that some of the observed associations (e.g., stress levels or breastfeeding duration) might not remain significant once such adjustments are applied. Future studies employing representative sampling and validated measures are needed to confirm and expand upon these results.

## Figures and Tables

**Table 1:** Study population characteristics.

	SARS-CoV-2	Control	All Respondents	Chi^2^ *p*-Value
**Number**	125	136	261	
**Age**				
18–25	1 (0.8%)	2 (1.5%)	3 (1.1%)	0.9414
26–35	66 (52.8%)	75 (55.1%)	141 (54.0%)	0.7981
36–45	57 (45.6%)	59 (43.4%)	116 (44.4%)	0.8138
46–55	1 (0.8%)	0 (0.0%)	1 (0.4%)	0.9663
**Education**				
Lower education	9 (7.2%)	11 (8.1%)	20 (7.7%)	0.9708
Higher education	116 (92.8%)	125 (91.9%)	241 (92.3%)	0.9708
**Living Environment**				
Rural	12 (9.6%)	15 (11.0%)	27 (10.3%)	0.8608
Urban	113 (90.4%)	121 (89.0%)	234 (89.7%)	0.8608
**Breastfeed during the Pandemic**				
No	0 (0.0%)	7 (5.1%)	7 (2.7%)	0.0287 *
Breastfeeding and formula	17 (13.6%)	24 (17.6%)	41 (15.7%)	0.467
Exclusively breastfed	108 (86.4%)	105 (77.2%)	213 (81.6%)	0.0792
**Breastfeeding Duration**				
<1 month	0 (0.0%)	5 (3.7%)	5 (1.9%)	0.0868
1–3 months	1 (0.8%)	8 (5.9%)	9 (3.4%)	0.0563
3–6 months	3 (2.4%)	9 (6.6%)	12 (4.6%)	0.1837
6–12 months	9 (7.2%)	17 (12.5%)	26 (10.0%)	0.2219
more than 1 year	112 (89.6%)	97 (71.3%)	209 (80.1%)	0.0004 ***

* *p* < 0.05, *** *p* < 0.001.

**Table 2:** Descriptive statistics for the emotional measures assessed.

	Control	SARS-CoV-2	All	Chi^2^_*p*_Value
I was in a good state	18/136 (13.2%)	9/125 (7.2%)	27/261 (10.3%)	0.163
Panic attacks	14/136 (10.3%)	16/125 (12.8%)	30/261 (11.5%)	0.660
Low zest for life	19/136 (14%)	14/125 (11.2%)	33/261 (12.6%)	0.627
Hopelessness about the future	29/136 (21.3%)	22/125 (17.6%)	51/261 (19.5%)	0.547
Low energy	29/136 (21.3%)	26/125 (20.8%)	55/261 (21.1%)	0.961
Unrealistic and excessive worries	32/136 (23.5%)	34/125 (27.2%)	66/261 (25.3%)	0.590
Insomnia	12/136 (8.8%)	18/125 (14.4%)	30/261 (11.5%)	0.224
I feared the possibility of my newborn or child becoming infected	81/136 (59.6%)	90/125 (72%)	171/261 (65.5%)	0.047 *
I feared for my life and my loved ones’ lives	58/136 (42.6%)	71/125 (56.8%)	129/261 (49.4%)	0.031 *
Panic related to the unknowns of the disease	68/136 (50%)	74/125 (59.2%)	142/261 (54.4%)	0.172
Loneliness	28/136 (20.6%)	23/125 (18.4%)	51/261 (19.5%)	0.772
Somatizations	15/136 (11%)	10/125 (8%)	25/261 (9.6%)	0.535
Tendency to isolate	42/136 (30.9%)	47/125 (37.6%)	89/261 (34.1%)	0.311
Tendency towards compulsive washing and disinfecting	46/136 (33.8%)	58/125 (46.4%)	104/261 (39.8%)	0.052
Sadness	31/136 (22.8%)	23/125 (18.4%)	54/261 (20.7%)	0.470

* *p* < 0.05.

## Data Availability

The raw data supporting the conclusions of this article will be made available by the authors upon request.

## Abbreviations

The following abbreviations are used in this manuscript:

WHO	World Health Organization
COVID-19	Coronavirus disease 2019
SARS-CoV-2	Severe acute respiratory syndrome coronavirus 2
